# Digital Platform for Continuous Monitoring of Patients Using a Smartwatch: Longitudinal Prospective Cohort Study

**DOI:** 10.2196/47388

**Published:** 2023-09-12

**Authors:** Kaio Jia Bin, Lucas Ramos De Pretto, Fábio Beltrame Sanchez, Fabio Pacheco Muniz De Souza e Castro, Vinicius Delgado Ramos, Linamara Rizzo Battistella

**Affiliations:** 1 Instituto de Medicina Física e Reabilitação Hospital das Clínicas Faculdade de Medicina da Universidade de São Paulo São Paulo Brazil

**Keywords:** smartwatch, digital health, telemedicine, wearable, telemonitoring, mobile health, General Data Protection Regulation, GDPR, Lei Geral de Proteção de Dados, LGPD, digital platform, clinical intervention, sensitive data, clinical trial, mobile phone

## Abstract

**Background:**

Since the COVID-19 pandemic, there has been a boost in the digital transformation of the human society, where wearable devices such as a smartwatch can already measure vital signs in a continuous and naturalistic way; however, the security and privacy of personal data is a challenge to expanding the use of these data by health professionals in clinical follow-up for decision-making. Similar to the European General Data Protection Regulation, in Brazil, the *Lei Geral de Proteção de Dados* established rules and guidelines for the processing of personal data, including those used for patient care, such as those captured by smartwatches. Thus, in any telemonitoring scenario, there is a need to comply with rules and regulations, making this issue a challenge to overcome.

**Objective:**

This study aimed to build a digital solution model for capturing data from wearable devices and making them available in a safe and agile manner for clinical and research use, following current laws.

**Methods:**

A functional model was built following the Brazilian *Lei Geral de Proteção de Dados* (2018), where data captured by smartwatches can be transmitted anonymously over the Internet of Things and be identified later within the hospital. A total of 80 volunteers were selected for a 24-week follow-up clinical trial divided into 2 groups, one group with a previous diagnosis of COVID-19 and a control group without a previous diagnosis of COVID-19, to measure the synchronization rate of the platform with the devices and the accuracy and precision of the smartwatch in out-of-hospital conditions to simulate remote monitoring at home.

**Results:**

In a 35-week clinical trial, >11.2 million records were collected with no system downtime; 66% of continuous beats per minute were synchronized within 24 hours (79% within 2 days and 91% within a week). In the limit of agreement analysis, the mean differences in oxygen saturation, diastolic blood pressure, systolic blood pressure, and heart rate were −1.280% (SD 5.679%), −1.399 (SD 19.112) mm Hg, −1.536 (SD 24.244) mm Hg, and 0.566 (SD 3.114) beats per minute, respectively. Furthermore, there was no difference in the 2 study groups in terms of data analysis (neither using the smartwatch nor the gold-standard devices), but it is worth mentioning that all volunteers in the COVID-19 group were already cured of the infection and were highly functional in their daily work life.

**Conclusions:**

On the basis of the results obtained, considering the validation conditions of accuracy and precision and simulating an extrahospital use environment, the functional model built in this study is capable of capturing data from the smartwatch and anonymously providing it to health care services, where they can be treated according to the legislation and be used to support clinical decisions during remote monitoring.

## Introduction

### Background

After the World Health Organization declared the COVID-19 pandemic on March 11, 2020 [1], with >470 million cases of infection and >6 million deaths confirmed [2], the digital transformation in health care worldwide has accelerated [3-5]. In this scenario, where the most frequent comorbidities are hypertension (55%), coronary artery disease and stroke (32%), and diabetes (31%) [6], monitoring vital signs such as oximetry, blood pressure (BP), and heart rate can be of paramount importance to monitor the evolution of patients infected by COVID-19. Therefore, wearable devices, such as smartwatches, are key actors in revolutionizing telemedicine [7-9] through mobile health and digital health (eHealth), allowing continuous and longitudinal health monitoring outside health care facilities [10].

Because of the ease of use of smartwatches, initially aimed at consumers concerned about their own health, several studies have shown interest in its application for remote monitoring [11] and as a tool for telemonitoring and early detection of respiratory symptoms [12,13], heart disease [14-17], and remote physician therapy [18]. Smartwatches can record clinical data in a way that feels organic and unobtrusive to the user, enabling the construction of a database that will facilitate, with the aid of artificial intelligence, the recognition of biomarkers capable of expanding the mechanisms of prediction, prevention, and health event intervention. There are reports in the literature discussing smartwatch data collection [19], with the most recent reports suggesting the possibility of early detection of COVID-19 [12,13] and atrial fibrillation [15-17]. In addition, wearable devices present a new perspective on patient monitoring with its continuous and naturalistic method of data collection without user action. In a cohort study involving >59,000 patients, it was found that BP measurements obtained through 24-hour monitoring were more informative about the risk of death from cardiovascular reasons than conventional measurements taken in the clinic [20].

However, for wearable devices to be usable in a clinical setting, a data processing model that respects legislation, privacy, and data security is necessary to enable the clinical use of data. In October 1995, through Directive 95/46/EC [21], the European Parliament approved the first regulation for the block with the legal concepts of data protection, which was definitively replaced in May 2018 by the General Data Protection Regulation [22], published on April 27, 2016. Inspired by the European General Data Protection Regulation, in Brazil, on August 14, 2018, the *Lei Geral de Proteção de Dados* (LGPD) [23] established rules and guidelines for the processing of personal data, including those used for patient care, such as those captured by smartwatches. Therefore, in any telemonitoring scenario, where patient information needs to reach the caregiver, there is a need to comply with rules and regulations, making this issue a challenge to overcome [24].

According to Article 12 of the LGPD [23], “Anonymized data will not be considered personal data for the purposes of this Law, except when the anonymization process to which they were submitted is reversed, using exclusively their own means, or when, with reasonable efforts, it can be reversed.” In this way, if the data collected are already anonymized, they can receive the appropriate treatment “for health guardianship, exclusively, in a procedure carried out by health professionals, health services, or health authorities” (LGPD, 2018, Articles 7 and 11) [23].

Therefore, if data from smartwatches are captured anonymously, with the user’s identification replaced by a random code, they can be transmitted via the Internet of Things (IoT) from any location to a cloud; from there, the data will be retransmitted to health services so that they can be integrated with their respective medical records, where they have the correct identification of the user through the random codes used in the transmission.

### Motivation

To enable the future of telemonitoring, early detection, and remote therapies, there is a need for an information processing model capable of collecting data from smartwatches, continuously and effectively, that respect legislation, privacy, and data security, where the accuracy and precision can be evaluated as well.

### Aim

This study aimed to build a digital solution model for capturing data from wearable devices and making them available in a safe and agile manner for clinical and research use, following current laws.

This study also aimed to analyze the reliability and accuracy of vital sign measurements (BP, heartbeat/min, and oxygen saturation) of the Samsung Galaxy Watch 4 (Samsung Group) with gold-standard measurement equipment (digital wrist BP device and finger oximeter).

## Methods

### Overview

A digital solution, named LIKA, was developed to support the entire clinical study; details on data transfer, data flow, data synchronization, and components and functionalities can be found in the preliminary report published earlier [25]. Its role is to guarantee anonymity, completeness, and reliability of the data collected, as well as to consolidate different sources of information input.

### Ethics Approval

This project was submitted to the Ethics and Research Committee of the Hospital das Clinicas, the Faculdade de Medicina of the University of São Paulo under number CAAE: 51711921.3.0000.0068 and Opinion number 4,975,512.

### Study Type

This was a single-center prospective study following the guidelines “Evaluating digital health products” from the UK Health Security Agency [26,27], with local adaptations for the Brazilian population in the designing and testing phase of a digital product.

### Wearable and Gold-Standard Device

Each volunteer was provided with a smartwatch (Samsung Galaxy 4). The gold-standard devices used in this study were a noninvasive BP monitor, G-TECH model GP400 (Agência Nacional de Vigilância Sanitária [ANVISA] registration number 80275319016) with 2 AAA batteries; a pulse oximeter for noncontinuous monitoring, AFK model YK009 (ANVISA registration number 81995169005); and polysomnography equipment, iCelera Nano Poli (ANVISA registration number 80884610001).

A smartphone (Samsung A52) was provided to volunteers who did not have a Samsung mobile phone.

### Data Setting

A total of 80 volunteers were selected from the Hospital das Clínicas Faculdade de Medicina da Universidade de São Paulo collaborators to use the smartwatch over 24 weeks, with daily visits by research monitors. Oxygen saturation and BP data from both volunteers’ smartwatch and the gold-standard devices were collected and registered to REDCap (Research Electronic Data Capture; Vanderbilt University) [28], to be used to analyze the reliability and agreement between the gold-standard device data and the smartwatch data. Table 1 shows how each data point is collected from the smartwatch, and the configuration used is detailed in the preliminary report published earlier [25].

**Table 1:** Information collected from the smartwatch for this study.

Data	Collection	Gold-standard comparation
Steps	Automatic	No
Flights of stairs	Automatic	No
Exercise time	Automatic	No
Sleep quality	Automatic	Yes
Heart rate	Automatic	Yes
Oxygen saturation during sleep	Automatic	No
Oxygen saturation	User action	Yes
Blood pressure	User action	Yes
Weight	User action	No
Height	User action	No
Quantity and type of liquid ingested	User action	No
Quantity and type of food ingested	User action	No

### Statistical Analysis

The following section describes the statistical methods used to analyze the data. When relevant, continuous data are numerically described by mean, SD, and quartiles, whereas categorical data are described by percentages. The gold-standard measurement values are assumed to be the true underlying values and are referred to as the “true value.”

### Sample Description

The sample was designed around 2 main groups of interest: volunteers who had a confirmed diagnosis of COVID-19 before recruitment (confirmed by a reverse transcription polymerase chain reaction test), referred to as the “COVID-19 group,” and volunteers who were not affected by COVID-19, referred to as the “non–COVID-19 group.” The volunteers were selected for the groups to have similar distributions of race, sex, and age.

### Preprocessing

To mitigate the influence of human errors in the analysis, values that lie outside the biological viability were excluded from the analysis. These values were as follows: values <60% and >100% were excluded for oxygen saturation, and values below <20 and >300 were excluded for BP (mm Hg) and heart rate (beats/min [bpm]). For data acquired continuously, only data acquired on weekdays (Monday to Friday) were considered for the analysis, as the daily activities on these days present less variability than those during weekends.

Furthermore, a subset of data was collected daily and in triplicate for the evaluation of agreement between the smartwatch and the respective gold-standard equipment. To better assess the performance of the devices, the triplicates were treated before the analysis to minimize outlandish or erroneous readings. To this end, for each device and each triplicate round, 3 readings were sorted in ascending order of value. A tolerance region (TR) is calculated as 10% of the value of the smallest region. If the distance between the largest and smallest values of the triplicate is smaller than the TR, all 3 values are considered valid and averaged before being used in the analysis. If the middle value distance to one of the extreme (largest or smallest) values is smaller than the TR, then the middle value and the corresponding extreme are averaged before the analysis, whereas the other extreme is considered invalid. If the distance of the middle value to both extremes is smaller than the TR, only the middle value is considered valid and used for the analysis. Finally, if none of the values fall within the TR of each other, the variation between them is assumed to be a valid occurrence, and all 3 readings are averaged before the analysis. In that way, only 1 value is output for each triplicate and only 1 value per day is used in the analysis.

### Device Agreement Analysis

To check how the smartwatch performs on each of the measurements of interest (heart rate, oxygen saturation, and BP), it is of value to compare it with a known gold-standard device. This comparison should evaluate how the difference between the devices is related to their magnitude while, in this case, considering the fact that multiple measurements were made on the same individual, which constitutes a repeated measure design. The analysis of choice, then, was the limits of agreement (LoA) for repeated measure [29], which calculates an interval around the “true value” (ie, the gold standard) in which one should expect 95% of the measurements made with the smartwatch to fall within. This approach is widely applied in the literature and is based on a fixed-effects model with a 1-way ANOVA test. To understand how the agreement varies with regard to the groups, the data were segmented and the LoA were separately calculated for each group and qualitatively compared, because the analysis was not readily applicable for group comparison. The LoA analysis assumes that there is no bias of measurement with regard to the within-volunteer magnitude, which has been checked for graphically. In addition, Bland-Altman plots were used to visualize the LoA. Finally, the percentages of records that fell within different regions around the “true value” are presented.

### Group Comparison

Data collected for device agreement were also used for comparing the 2 groups. Each volunteer had their measurements across visits averaged for both the gold-standard device data and the smartwatch data. Then, the mean value of each group was compared using the 2-tailed *t* test for independent samples. The group comparison was carried out separately for the gold-standard device data and the smartwatch data.

### Continuous Data Analysis

The smartwatch continuously collected heart rate data from volunteers during their day-to-day activities, and it is noteworthy to check whether the heart rate behavior was different between the groups. To that end, one needs to consider not only the influence of the repeated measures design (as the same volunteer is evaluated on multiple days) but also the fact that there is an autocorrelation aspect, as the heart rate measured at time point t=0 is expected to be closer to that measured consecutively at time point t=1 than to the one measured at an arbitrary time point t=n later on. To accommodate these limitations, a profile analysis based on multivariate ANOVA (MANOVA) was conducted on the data. This approach provides the answer to three different hypotheses: (1) the level hypothesis, which checks whether the baseline heart rate differs across the groups (between-volunteer effects of MANOVA); (2) the flatness hypothesis, which tests if the heart rate varies across time (within-volunteer main effects); and (3) the parallel hypothesis, which responds to the main question of which the behavior of the heart rate across time differs across the groups (interaction term). For use of this analysis, the data of each volunteer were first averaged per hour and then across the days of measurement, resulting in a single vector of 24 positions (1 for each hour) for each volunteer. For statistical significance, *F* test was used with Hotelling T² and Wilks Λ. The results were considered statistically significant if *P*<.05. Plots of the average 24-hour curve of the heart rate of each group were used to visualize the results.

### Sleep Data Analysis

To evaluate sleep data quality, volunteers were asked to spend the night at Instituto de Medicica Física e Reabilitação under the supervision of a polysomnography technician, where data gathered from a polysomnography equipment (iCelera Nano Poli) was used as a gold standard to compare against the sleep data collected automatically and simultaneously by the smartwatch.

Because of operational difficulties, there was a delay in the delivery of the polysomnography equipment as well as in training the polysomnography technician team. This delay led to the late start of data acquisition, and only 8 volunteers could be evaluated in this study.

However, the data recorded by the smartwatch did not follow the same methodology as the gold-standard equipment. The former started sleep data recording when the first sleep stage [30] is reached, whereas the latter started when the volunteer lies in bed. Furthermore, the gold-standard equipment recorded data continuously at every 30 seconds, whereas the smartwatch only recorded summarized data (start and end time of each sleep stage observed). Because of these facts and the limited number of volunteers available for analysis, the sleep data were presented only through descriptive statistics and compared qualitatively. In addition, the gold-standard equipment recorded 5 different stages of sleep (awake, N1, N2, N3, and rapid eye movement [REM]), and the smartwatch recorded 4 stages (awake, light sleep, deep sleep, and REM). For comparison purposes, the N1 and N2 stages of the gold-standard measurement were combined into a single stage, equivalent to the “light sleep” stage of the smartwatch, whereas the N3 stage was considered equivalent to the “deep sleep” stage, as recommended by the available literature [31].

The metric of “time in bed” (TIB) considers the total time of when the volunteer lied in bed up to when the volunteer is fully awake (according to the device). For the gold-standard measurement, these were the times of start and end of the report. Because the smartwatch did not record the time when the patient lies in bed, the time obtained from the gold-standard measurement was used instead. Thus, the TIB for the smartwatch goes from the start time of the gold-standard measurement up to the start time of the last “awake” stage recorded for that session.

Therefore, the sleep data are described in this study by the summary statistics of the following measurements: total sleep time (TST); sleep time of each stage; sleep efficiency (SE), which is the ratio of the TST to the TIB; sleep latency, which is the time from the beginning of the TIB to the first recorded sleep stage; and wake after sleep onset (WASO), which is the total time awake after the first sleep stage.

### Alert System Measurement

The following triggers were defined for the BP alerts based on the manufacturer’s recommendations [32]:

- Systolic BP
  ≤70 mm Hg
  >180 mm Hg
- Diastolic BP
  ≤40 mm Hg
  >120 mm Hg

Every time one of these events was triggered by the digital platform, the value, date, hour, and volunteer’s identifier were displayed on the dashboard with the intention of monitoring the use of the smartwatch by the volunteers. Furthermore, considering that the normal oxygen levels in a pulse oximeter usually range from 95% to 100% and hypoxemia is an oxygen saturation of >90% [33], the digital platform was set up to alert when the value reaches <88%. Similarly, considering that the diagnosis of sinus bradycardia requires an electrocardiogram showing a normal sinus rhythm at a rate ≤60 bpm [34], the digital platform was set up to alert when the heart rate fell ≤40 bpm.

### User Experience Survey Form

A satisfaction survey with a focus on the incorporation of technology, based on the paper published by Nelson et al [35], was applied for all volunteers at the end of the clinical follow-up. The question chosen was “When using the smartwatch, it almost feels like it is incorporated into the body,” and translated for our population in Portuguese as “Ao usar o relógio inteligente, sinto que ele está incorporado ao meu corpo.”

## Results

### Digital Solution: LIKA

A complete system of data capture, transmission through the IoT, retransmission to the hospital, and visualization of information was built to support the study and named as LIKA. The system received its first volunteer for monitoring on February 25, 2022 [25].

### Volunteers

A total of 122 volunteers were selected for the study, of whom 42 were replaced by new volunteers with the same sociodemographic profile (Table 2). The 80 selected volunteers, divided into 2 groups (Table 3), were monitored for 35 weeks, from February 25, 2022, to October 21, 2022. Data from dropped out volunteers (465 records) were excluded (Table 4). In total, there were 13,156 days of follow-up for the 80 volunteers, 43 of whom completed 24 weeks of follow-up as initially planned (Table 5).

**Table 2:** Volunteer recruitment data (N=122).

	Volunteers, n (%)
Recruited for research	122 (100)
Did not show up at the interview	17 (13.9)
Did not meet the requirements during the interview	10 (8.2)
Dropped out after inclusion in the study	12 (9.8)
Dropped out after beginning telemonitoring	3 (2.5)
Total volunteers in the study	80 (65.6)

**Table 3:** Volunteer grouping based on previous diagnosis of COVID-19, sex, age group, and race.

Characteristic		Non–COVID-19 group (n=40), n (%)	COVID-19 group (n=40), n (%)	Total (N=80), n (%)
**Sex**				
	Female	30 (75)	30 (75)	60 (75)
	Male	10 (25)	10 (25)	20 (25)
**Race**				
	Asian	3 (8)	3 (8)	6 (8)
	Black	3 (8)	2 (5)	5 (6)
	Indigenous	0 (0)	1 (3)	1 (1)
	White	17 (43)	17 (43)	34 (43)
	Mixed race	17 (43)	17 (43)	34 (43)
**Age group (years)**				
	22-39	14 (35)	14 (35)	28 (35)
	40-59	21 (53)	21 (53)	42 (53)
	≥60	5 (13)	5 (13)	10 (13)

**Table 4:** Total number of users by weeks of project.

Week	New users (n=83), n (%)	Dropout users (n=3), n (%)	Total users (n=80), n (%)
1-4	15 (18)	0 (0)	15 (19)
5-8	35 (42)	3 (100)	47 (59)
9-12	17 (20)	0 (0)	64 (80)
13-16	12 (14)	0 (0)	76 (95)
17-20	2 (2)	0 (0)	78 (98)
21-24	1 (1)	0 (0)	79 (99)
25	1 (1)	0 (0)	80 (100)
26-35	0 (0)	0 (0)	80 (100)

**Table 5:** Number of volunteers per follow-up time in weeks and days.

Follow-up weeks	Volunteers (n=80), n (%)	Total days (n=13,156), n (%)
<8	0 (0)	0 (0)
8-11	4 (5)	282 (2.1)
12-15	4 (5)	415 (3.2)
16-19	10 (12.5)	1276 (9.7)
20-23	19 (23.8)	2945 (22.4)
24-27	26 (32.5)	4742 (36)
28-30	17 (21.3)	3496 (26.6)

### Data Collection

In total, 11,229,796 records were captured: 10,510,351 for continuous heart rate; 289,749 for heart rate; 25,212 for oxygen saturation; 16,123 for BP; 12,410 for sleep count (marks the beginning and end of sleep); and 375,951 for sleep intensity (marks the beginning and end of each phase of sleep; Table 6). A few records of vital signs did fall outside the range of normative values, and to monitor these, the alert system within our framework was used. In total, 1312 alerts were generated (Table 7). Furthermore, to assess whether the platform can be used by the clinical team as a digital health care solution, it is very important that the data be available on the digital platform as soon as possible after being recorded by the smartwatch. The time interval between the moment of recording the continuous bpm and the moment it is available on the digital platform depends on the synchronization between the manufacturer’s health app and the developed digital solution. As shown in Table 8, less than 5% of the records were synchronized within the first 2 hours of interval time. On the other hand, most data (6,974,213/10,510,351, 66.36%) were synchronized within 24 hours in either group. Furthermore, only <10% of the data were synchronized after 7 days.

**Table 6:** Total data record by type.

Data type count	Total records (n=11,229,796), n (%)
Continuous heart bpm^a^	10,510,351 (93.59)
Heart bpm	289,749 (2.58)
Oxygen saturation	25,212 (0.22)
Blood pressure	16,123 (0.14)
Sleep	12,410 (0.11)
Sleep intensity	375,951 (3.35)

^a^bpm: beats per minute.

**Table 7:** Type of alerts, amount, and index per category^a^.

Type of alerts	Amount (n=1312), n (%)	Volunteers (n=80), n (%)
BP^b^ diastolic maximus (≥120)	7 (0.53)	3 (3.8)
BP diastolic minima (≤40)	0 (0)	0 (0)
BP systolic maximus (≥180)	4 (0.3)	2 (2.5)
BP systolic minima (≤70)	0 (0)	0 (0)
Low oxygen saturation (<88)	1200 (91.46)	67 (83.8)
Low heart rate (<40)	21 (1.6)	5 (6.3)

^a^Total: all 1312 alerts and 69 (86%) out of 80 volunteers.

^b^BP: blood pressure.

**Table 8:** Synchronization time of continuous beats per minute.

Synchronization time	Amount (n=10,510,351), n (%)	Accumulated (%)
<1 h	40,657 (0.39)	0.39
1-2 h	455,285 (4.33)	4.72
3-6 h	1,429,176 (13.6)	18.32
7-12 h	2,094,408 (19.93)	38.24
13-24 h	2,954,687 (28.11)	66.36
1-2 d	1,366,680 (13)	79.36
3-7 d	1,278,007 (12.16)	91.52
8-14 d	519,138 (4.94)	96.46
>14 d	372,313 (3.54)	100

### Sleep Data

Data were collected from 8 volunteers for this study. Table 9 lists the TIB for each volunteer. The summary metrics of TST, SE, sleep latency, WASO, and the subtotals of each sleep stage for each volunteer and each device are listed in Table 10. The gold standard used was iCelera Nano Poli Polysomnography equipment.

From Table 9, it is notable that the smartwatch generally overestimates the TIB, perhaps because the smartwatch needs a longer period of activity to consider the volunteer fully awake. This is especially notable in the data of volunteers 1 and 5, where there was a large discrepancy between the smartwatch and the gold-standard measurements.

The WASO differences observed in Table 10 may have the same underlying cause. As the gold-standard device’s reports are evaluated every 30 seconds, it could be the case of small periods of the volunteer being awake that are captured by the polysomnography device, but are missed or, at least, not recorded by the smartwatch, which would explain why the wearable device undershoots the WASO in 7 of the 8 volunteers. Analogously, the sleep latency tends to be larger for the smartwatch, as brief periods of light sleep may be missed—even more so as this is the beginning of sleep. Nevertheless, the metrics are not overwhelmingly divergent between the devices, especially the SE.

The sleep stages were discriminated for each volunteer and device in Table 11. Awake times for some volunteers presented large deviations between the equipment, perhaps for the same reasons mentioned earlier. Nevertheless, the light sleep and deep sleep times presented by the smartwatch are generally close to the gold-standard times. REM, on the other hand, tends to be largely overestimated by the smartwatch, and the underlying causes should be further investigated (such as what is considered REM by the smartwatch algorithm).

**Table 9:** Time in bed (TIB) values recorded for each volunteer.

Device			TIB begin (date; time in hours)^a^		TIB end (date; time in hours)^a^		TIB (h)
**Volunteer 1**							
	Gold standard	August 10, 2022; 20:21:37		August 11, 2022; 04:58:47		08:37:10	
	Smartwatch	August 10, 2022; 20:21:37		August 11, 2022; 06:25:00		10:03:23	
**Volunteer 2**							
	Gold standard	August 16, 2022; 21:11:45		August 17, 2022; 04:46:08		07:34:23	
	Smartwatch	August 16, 2022; 21:11:45		August 17, 2022; 04:48:00		07:36:15	
**Volunteer 3**							
	Gold standard	June 15, 2022; 22:52:34		June 16, 2022; 06:11:14		07:18:40	
	Smartwatch	June 15, 2022; 22:52:34		June 16, 2022; 06:47:00		07:54:26	
**Volunteer 4**							
	Gold standard	August 4, 2022; 21:36:38		August 5, 2022; 06:00:48		08:24:10	
	Smartwatch	August 4, 2022; 21:36:38		August 5, 2022; 06:36:00		08:59:22	
**Volunteer 5**							
	Gold standard	September 21, 2022; 20:07:38		September 22, 2022; 03:32:48		07:25:10	
	Smartwatch	September 21, 2022; 20:07:38		September 22, 2022; 05:11:00		09:03:22	
**Volunteer 6**							
	Gold standard	August 24, 2022; 21:53:56		August 25, 2022; 05:24:33		07:30:37	
	Smartwatch	August 24, 2022; 21:53:56		August 25, 2022; 05:40:00		07:46:04	
**Volunteer 7**							
	Gold standard	August 4, 2022; 21:51:54		August 5, 2022; 05:23:34		07:31:40	
	Smartwatch	August 4, 2022; 21:51:54		August 5, 2022; 06:04:00		08:12:06	
**Volunteer 8**							
	Gold standard	August 24, 2022; 21:10:57		August 25, 2022; 06:06:25		08:55:28	
	Smartwatch	August 24, 2022; 21:10:57		August 25, 2022; 06:15:00		09:04:03	

^a^The date and time for the beginning of TIB were based on the gold-standard device report.

**Table 10:** Descriptive summary statistics of sleep data for each volunteer.

Device		First sleep stage (date; time in hours)	Sleep latency (h)	TST^a^ (h)	SE^b^ (%)	WASO^c^ (h)
**Volunteer 1**						
	Gold standard	August 10, 2022; 21:14:07	00:52:30	06:51:30	79.57	00:58:00
	Smartwatch	August 10, 2022; 21:51:00	01:29:23	08:07:00	80.71	00:27:00
**Volunteer 2**						
	Gold standard	August 16, 2022; 22:48:17	01:36:32	04:48:00	63.38	01:35:30
	Smartwatch	August 16, 2022; 22:36:00	01:24:15	05:30:00	72.33	00:42:00
**Volunteer 3**						
	Gold standard	June 15, 2022; 23:22:34	00:30:00	06:22:00	87.08	00:28:00
	Smartwatch	June 15, 2022; 23:42:00	00:49:26	06:05:00	76.93	01:00:00
**Volunteer 4**						
	Gold standard	August 4, 2022; 22:29:38	00:53:00	05:52:00	69.82	01:40:00
	Smartwatch	August 5, 2022; 00:11:00	02:34:22	05:47:00	64.33	00:38:00
**Volunteer 5**						
	Gold standard	September 21, 2022; 20:35:08	00:27:30	06:06:30	82.33	00:52:30
	Smartwatch	September 21, 2022; 22:07:00	01:59:22	06:24:00	70.67	00:40:00
**Volunteer 6**						
	Gold standard	August 24, 2022; 22:21:06	00:27:10	06:05:00	81.00	01:03:00
	Smartwatch	August 24, 2022; 22:07:00	00:13:04	06:38:00	85.40	00:45:00
**Volunteer 7**						
	Gold standard	August 4, 2022; 22:33:54	00:42:00	05:34:00	73.95	01:18:00
	Smartwatch	August 4, 2022; 23:19:00	01:27:06	05:28:00	66.65	01:17:00
**Volunteer 8**						
	Gold standard	August 24, 2022; 21:36:15	00:25:18	07:51:00	87.96	00:37:00
	Smartwatch	August 24, 2022; 21:33:00	00:22:03	07:46:00	85.65	00:56:00

^a^TST: total sleep time.

^b^SE: sleep efficiency.

^c^WASO: wake after sleep onset.

**Table 11:** Record of total time for each sleep stage.

Device		Awake (h)	Light sleep (h)	Deep sleep (h)	REM^a^ (h)
**Volunteer 1**					
	Gold standard	01:50:30	05:11:30	01:20:30	00:19:30
	Smartwatch	00:27:00	04:25:00	01:22:00	02:20:00
**Volunteer 2**					
	Gold standard	03:11:30	03:50:30	00:43:30	00:14:00
	Smartwatch	00:42:00	02:55:00	00:51:00	01:44:00
**Volunteer 3**					
	Gold standard	00:58:00	04:34:00	01:29:30	00:18:30
	Smartwatch	01:00:00	03:28:00	00:41:00	01:56:00
**Volunteer 4**					
	Gold standard	02:33:00	04:46:00	00:52:00	00:14:00
	Smartwatch	00:38:00	03:50:00	00:32:00	01:25:00
**Volunteer 5**					
	Gold standard	01:20:00	05:07:30	00:23:00	00:36:00
	Smartwatch	00:40:00	03:22:00	01:20:00	01:42:00
**Volunteer 6**					
	Gold standard	01:30:00	03:48:00	01:12:00	01:05:00
	Smartwatch	00:45:00	03:00:00	01:53:00	01:45:00
**Volunteer 7**					
	Gold standard	02:00:00	04:08:00	01:02:30	00:23:30
	Smartwatch	01:17:00	03:10:00	01:01:00	01:17:00
**Volunteer 8**					
	Gold standard	01:02:00	05:14:30	01:15:00	01:21:30
	Smartwatch	00:56:00	03:41:00	01:13:00	02:52:00

^a^REM: rapid eye movement.

### User Experience

To evaluate the user experience, a satisfaction questionnaire was administered at the end of the volunteers’ participation. Three questions regarding the use of the smartwatch were asked using a Likert scale. The responses are presented in Tables 12-14. It is notable that most users felt comfortable using the smartwatch, with 69% (47/68) agreeing at least partially that the device felt like it was part of their own body, whereas the rejection of that sentiment was only 16% (11/68). In addition, most of the volunteers (38/68, 56%) were interested in acquiring a smartwatch after the study. When not considering the ones who already had a wearable device (11/68, 16%), a total of 38 volunteers who were introduced to using a smartwatch during the study said they were considering buying one, which indicates a good experience. Finally, 93% (63/68) of the volunteers felt like recommending the purchase of a smartwatch to friends or family (answers 7-10), with 67% (39/58) strongly recommending it (answers 9 and 10), and a median recommendation of 9, which is another good indicator of the easy and unobtrusive use of the device in their day-to-day life.

**Table 12:** User experience query about the feeling of using the smartwatch (n=68).

When using the smartwatch, it almost feels like it is incorporated in my body.	Values, n (%)
I fully agree	19 (28)
I partially agree	28 (41)
I neither agree nor disagree	10 (15)
I partially disagree	6 (9)
I strongly disagree	5 (7)

**Table 13:** User experience queries about feelings of ownership (n=68).

After returning the Galaxy Watch 4 smartwatch	Values, n (%)
You already had one of the same brand to use.	2 (3)
You already had one from another brand to use.	9 (13)
You did not have any to use, but you decided you are going to buy the same or a similar one.	38 (56)
You did not have any to use, and you are not going to buy any similar ones to use.	19 (28)

**Table 14:** User experience query about the recommendation to a family member (n=68)^a^.

Considering your experience using the smartwatch during the research project, how likely are you to recommend its purchase to a family member or friend on a scale of 1 to 10?	Values, n
1	3
2	0
3	1
4	0
5	1
6	0
7	3
8	21
9	14
10	25

^a^The median was 9.

### LoA Analysis

To evaluate the performance of the smartwatch, a comparison to a known gold-standard device was made. The LoA analysis was performed separately for each measure of interest.

**Figure 1:**
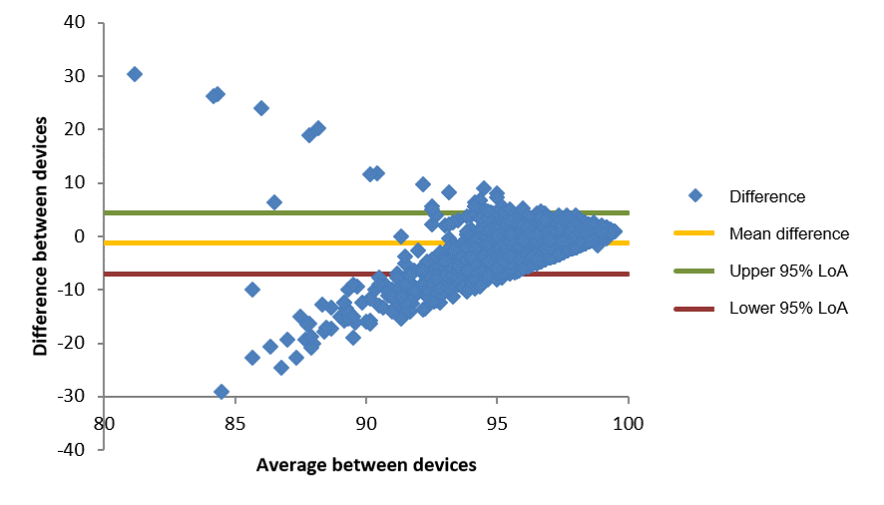
Bland-Altman plot of oxygen saturation differences (%) comparing the smartwatch and the AFK YK009 oximeter measurements. LoA: limits of agreement.

For oxygen saturation, the smartwatch was compared with the gold-standard AFK YK009 Pulse Oximeter. A total of 4521 pairs of records (after exclusion and before processing) were analyzed. The differences are computed as the gold-standard measurement subtracted from the smartwatch measurement, so a positive value indicates that the smartwatch overestimated the value, whereas a negative value indicates an underestimation. The Bland-Altman plot is presented in Figure 1, and the LoA values are listed in Table 15.

The fan-shaped distribution presented in Figure 1 is because the measurement was inherently capped at 100%. The calculated differences for measurements that lie close to the maximum value (which were also closer to the normal physiological range) were bound to lower or negative values. Nevertheless, the LoA established reasonable agreement, with a lower limit of −6.9% and an upper limit of 4.4%, that is, one can expect that in 95% of cases, the error of the oxygen saturation measured by the smartwatch had a magnitude of, at most, slightly <7%. This LoA was also calculated across different ranges of the “true value” (as measured by the gold-standard device) by selecting only those cases in which the gold-standard measurement was within specific regions (100%-98%, 100%-95%, and 100%-90%). The LoA was very stable across all ranges, with a lower limit always close to −7% and an upper limit <2%. The mean difference (also called bias) was found to be −1.28%, suggesting that the smartwatch, in general, tends to underestimate the saturation of oxygen.

Finally, by segmenting the Bland-Altman plot in increasing intervals around 0 (in the y axis), one can compute how many of the cases are within a specific magnitude of error. As reported in Table 16, it is notable that the smartwatch missed the “true value” by <1% of oxygen saturation in 43.97% (1988/4521) of the cases, whereas the error was <3% of oxygen saturation in 86.09% (3892/4521) of all the measurements performed.

**Table 15:** Limits of agreement (LoA) calculated for oxygen saturation.

Oxygen saturation LoA	Values (%)
Mean difference	−1.28
Upper 95% limit	4.399
Lower 95% limit	−6.960

**Table 16:** Percentage of oxygen saturation error (difference between smartwatch and gold-standard measurements) within specific ranges.

Range	Oxygen saturation error distribution (%)
Within 1%	43.97
Within 2%	71.53
Within 3%	86.09

The same procedure of analysis was repeated for the BP measurements. A total of 4480 pairs of records were available for the analysis. However, the analysis was further divided as the diastolic and systolic BP were evaluated separately from each other, because their performances in relation to the gold-standard measurements were not assumed to be related. The gold-standard device used for BP monitoring is the G-TECH GP400 digital BP monitor.

**Figure 2:**
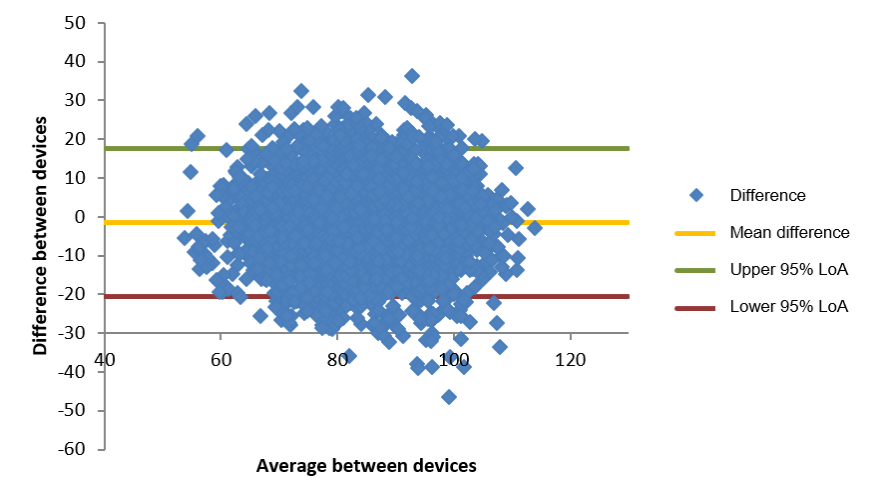
Bland-Altman plot of diastolic blood pressure differences (mm Hg) comparing the smartwatch and the G-TECH GP400 blood pressure monitor measurements. LoA: limits of agreement.

The Bland-Altman plot for diastolic BP is presented in Figure 2. The dispersion of data points seems concentrated in a specific region because of the underlying distribution of the “true value.” However, the errors are uniformly distributed in that region, with 95% LoA range of −20.5 mm Hg to 17.7 mm Hg, as indicated in Table 17. Similar to what was observed in the oxygen saturation case, this LoA range is fairly stable across different ranges of the “true value.” The mean difference observed is close to 0 (−1.399 mm Hg), which further reinforces the notion of a uniform spread of errors.

By, once again, segmenting the Bland-Altman plot into different regions around 0, it is possible to check the percentage of cases within a specific error magnitude. As seen in Table 18, 42.34% (1897/4480) of the readings were within 5 mm Hg of the “true value,” whereas 88.21% (3952/4480) were within 15 mm Hg. This is in accordance with the calculated 95% LoA, which indicates an error of, at most, 20.5 mm Hg in 95% of cases.

**Figure 3:**
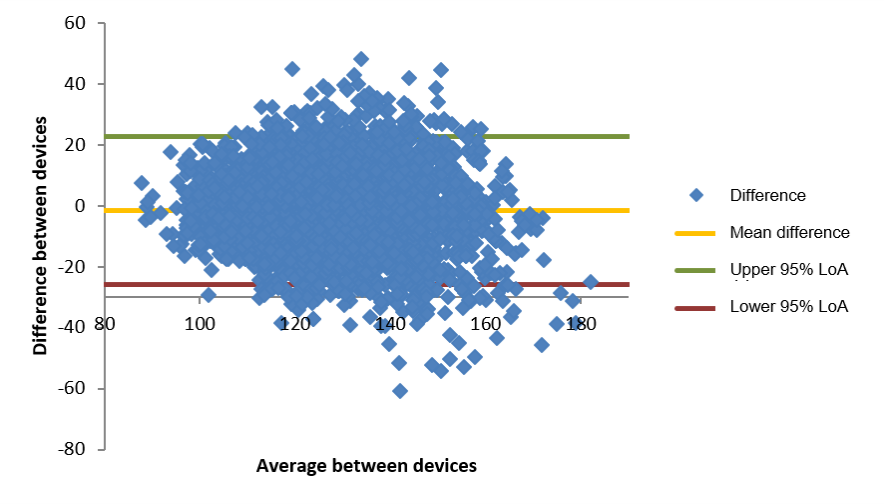
Bland-Altman plot of systolic blood pressure differences (mm Hg) comparing the smartwatch and the G-TECH GP400 blood pressure monitor measurements. LoA: limits of agreement.

The same analysis was repeated for systolic BP readings with similar findings. Figure 3 shows a dispersion concentrated in the expected region (according to the normative physiological range). The 95% LoA region (Table 19) was slightly larger than that for diastolic BP, ranging from −25.7 mm Hg to 22.7 mm Hg. Again, this LoA region was found to be stable across different sections of the “true value.” The bias was close to 0 at −1.5 mm Hg.

The distribution of errors also indicates a performance slightly worse than that observed for diastolic BP, with just <80% of the readings within 15 mm Hg of the “true value,” as presented in Table 20.

**Figure 4:**
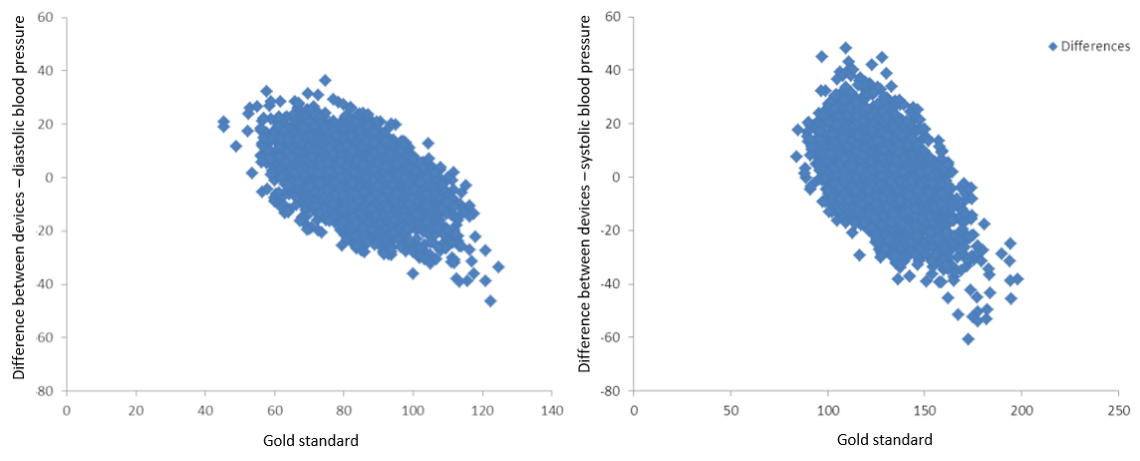
Distribution of smartwatch errors as a function of the respective gold-standard device value (true value). A linear trend was observed for both diastolic and systolic blood pressure readings. All axes are in mm Hg.

In addition, it is notable that there is an apparent linear behavior of the errors with the “true value,” as observed in Figure 4, for both diastolic and systolic BP. Linear regression analysis indicated that both distribution have similar slopes (−0.39 for diastolic BP and −0.36 for systolic BP), while the error seems to be centered on 82 mm Hg for the diastolic BP and it was close to 124 mm Hg for the systolic BP. This seems to suggest that the smartwatch is calibrated around these values and linearly deviates from the “true value” across the observed range.

**Table 17:** Limits of agreement (LoA) calculated for diastolic blood pressure (BP; mm Hg).

LoA for diastolic BP	Values (mm Hg)
Mean difference	−1.399
Upper 95% limit	17.713
Lower 95% limit	−20.517

**Table 18:** Percentage of diastolic blood pressure (BP) error (difference between the smartwatch and gold-standard measurements) within specific ranges.

Range	Diastolic BP error distribution (%)
Within 5 mm Hg	42.34
Within 10 mm Hg	71.67
Within 15 mm Hg	88.21

**Table 19:** Limits of agreement (LoA) calculated for systolic blood pressure (BP; mm Hg).

LoA for systolic BP	Values (mm Hg)
Mean difference	−1.536
Upper 95% limit	22.708
Lower 95% limit	−25.780

**Table 20:** Percentage of systolic blood pressure (BP) error (difference between the smartwatch and gold-standard measurements) within specific ranges.

	Systolic BP error distribution (%)
Within 5 mm Hg	35.69
Within 10 mm Hg	61.96
Within 15 mm Hg	79.35

A naive correlation analysis between the results obtained by both equipment shows a reasonable correlation coefficient, with *r*=0.60 for diastolic BP measurements and *r*=0.67 for systolic BP measurements. However, this result should be interpreted in light of a skewed distribution of BP values (mostly within the normative range), and also, because the measurement pairs are not independent, with repeated measures for each volunteer.

**Figure 5:**
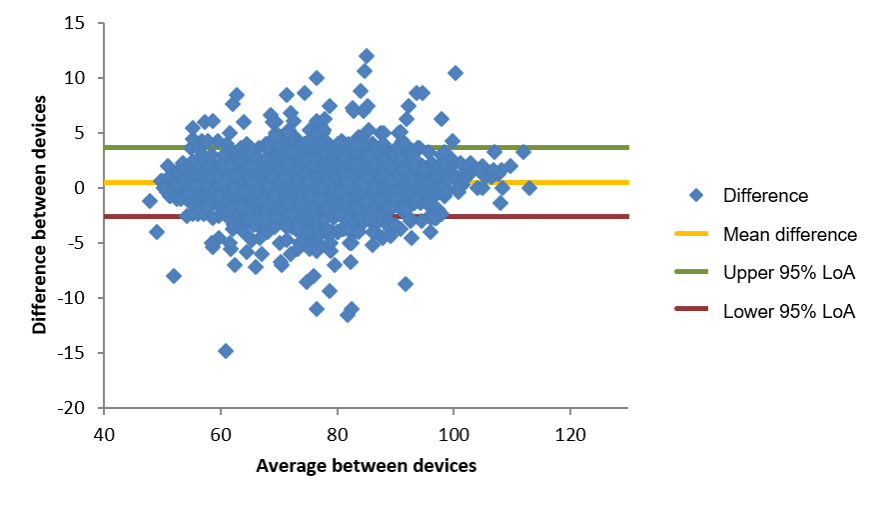
Bland-Altman plot of heart rate differences (beats/min) comparing the smartwatch and G-TECH GP400 blood pressure monitor measurements. LoA: limits of agreement.

Finally, the heart rate (measured in bpm) was evaluated against the gold-standard device measurement. The device used as the gold standard was the same as that used for BP measurements, the G-TECH GP400 digital BP monitor. In total, 4480 pairs of readings were analyzed. Compared with the former cases, the heart rate measurements present a much flatter distribution of errors across the observed values, which are in good agreement with the gold-standard measurements, as presented in Figure 5. The calculated 95% LoA region (Table 21) corroborates to the flatter distribution of errors, ranging from −2.54 bpm to 3.68 bpm. The bias averaged at less than 1 bpm.

The calculated LoA was uniform across different ranges of the “true value.” In addition, the distribution of errors across different segmentations of the Bland-Altman plot further reinforced the good agreement of the smartwatch measurements, with >60% of the readings within 1 bpm of the gold-standard value and just <95% of the cases within 3 bpm of the “true value”—as was expected from the 95% LoA, which indicates a maximum expected error of around 3.7 bpm. The results are listed in Table 22.

**Table 21:** Limits of agreement (LoA) calculated for heart rate (beats/min [bpm]).

LoA for heart rate	Value (bpm)
Mean difference	0.566
Upper 95% limit	3.680
Lower 95% limit	−2.548

**Table 22:** Percentage of heart rate error (difference between the smartwatch and gold-standard measurements) within specific ranges.

Range	Heart rate error distribution (%)
Within 1 bpm^a^	62.41
Within 2 bpm	87.16
Within 3 bpm	94.35

^a^bpm: beats per minute.

### LoA: Group Comparisons

The LoA procedure was applied separately to each group of the study. Although there is no statistical test to check for differences in the LoA, these results should be evaluated with regard to clinical relevance rather than a statistical one.

The results for all metrics for both groups are listed in Table 23, where the sample value indicated refers to the pairs of measurements (smartwatch and gold standard).

**Table 23:** Limit of agreement (LoA) analysis performed on each group individually^a^.

Measurement for each group		Values (n=4521), n (%)	Bias, mean difference (SD; 95% LoA)
**Oxygen saturation**			
	COVID-19	2363 (52.27)	−1.260 (2.890; 4.402 to −6.923)
	Non–COVID-19	2158 (47.73)	−1.304 (2.913; 4.407 to −7.015)
**Diastolic BP^b^**			
	COVID-19	2344 (52.32)	−0.968 (9.637; 17.921 to −19.858)
	Non–COVID-19	2136 (47.68)	−1.873 (9.876; 17.489 to −21.230)
**Systolic BP**			
	COVID-19	2344 (52.32)	−1.172 (12.613; 23.549 to −25.894)
	Non–COVID-19	2136 (47.68)	−1.934 (12.109; 21.800 to −25.669)
**Heart rate**			
	COVID-19	2344 (52.32)	−0.493 (1.595; 3.621 to −2.637)
	Non–COVID-19	2136 (47.68)	−0.646 (1.579; 3.741 to −2.448)

^a^LoA was calculated for each variable of interest. In all cases, the LoA was similar across the groups.

^b^BP: blood pressure.

For all variables tested, the LoA region was very similar in both the groups. The behavior observed in the overall LoA was replicated in the group analysis; the heart rate provided the narrower region, whereas the systolic BP presented the largest range. The smartwatch performance does not seem to be affected by the fact that the volunteer had a diagnosis of COVID-19.

### Daily Data: Group Comparisons

The data used to evaluate device agreement were also used to compare the groups themselves to verify whether there was any observable difference in the variables monitored.

The mean of each group for each variable, as recorded by the smartwatch, is listed in Table 24. The 2-tailed *t* test *P* values were adjusted for multiple comparisons.

Through smartwatch readings, it is not possible to find any difference between the volunteers who had COVID-19 and those who were not infected. Therefore, the small variations observed in the means may be because of sampling noise or a natural intrasample variation.

Confirming these results, the readings from the gold-standard devices also showed no statistically significant differences, as presented in Table 25.

The same behavior was observed for both equipment, and statistical significance was not achieved. Nevertheless, this result is unsurprising, as the volunteers in the COVID-19 group not only recovered from the infection, but were already back to their daily working routine, so no major symptoms or major complications from “post–COVID-19 condition” were expected.

**Table 24:** Group analysis of smartwatch readings.

Smartwatch reading	Non–COVID-19 group, mean^a^ (SD)	COVID-19 group, mean^a^ (SD)	*P* value^b^
Oxygen saturation (%)	95.930 (0.972)	96.253 (1.165)	.19
Heart rate (bpm^c^)	74.490 (8.222)	74.300 (7.154)	.91
Systolic blood pressure (mm Hg)	125.344 (12.175)	128.219 (12.660)	.31
Diastolic blood pressure (mm Hg)	82.640 (9.033)	85.004 (8.322)	.23

^a^The mean values were calculated from the averages of the volunteers.

^b^The *P* values were obtained from independent samples’ 2-tailed *t* tests. No statistically significant difference was found.

^c^bpm: beats per minute.

**Table 25:** Group analysis of the gold-standard readings.

Gold-standard reading	Non–COVID-19 group, mean^a^ (SD)	COVID-19 group, mean^a^ (SD)	*P* value^b^
Oxygen saturation (%)	97.366 (0.872)	97.475 (0.891)	.59
Heart rate (bpm^c^)	73.840 (8.206)	73.709 (7.111)	.94
Systolic blood pressure (mm Hg)	127.765 (12.673)	129.648 (12.972)	.52
Diastolic blood pressure (mm Hg)	84.730 (8.838)	85.050 (8.817)	.51

^a^The mean values were calculated from the averages of the volunteers.

^b^The *P* values were obtained from independent samples’ 2-tailed *t* tests. Similar to the smartwatch results, no statistically significant differences were observed.

^c^bpm: beats per minute.

### Continuous Monitoring: Group Comparisons

Similar to the previous section, the main interest involving continuous monitoring is not one regarding the smartwatch itself; its ability to monitor heart rate is proved by the LoA analysis. Rather, the interest lies in the volunteers themselves, whether the heart rate activity along the day presents any difference between those who had and those who did not have COVID-19. In that sense, the smartwatch is a unique tool that enables passive monitoring of patients without causing discomfort to the user.

Volunteers were monitored during the whole day, and only weekdays were included in the analysis. The measurements were averaged over an hour. In total, 8,041,871 records were analyzed.

**Figure 6:**
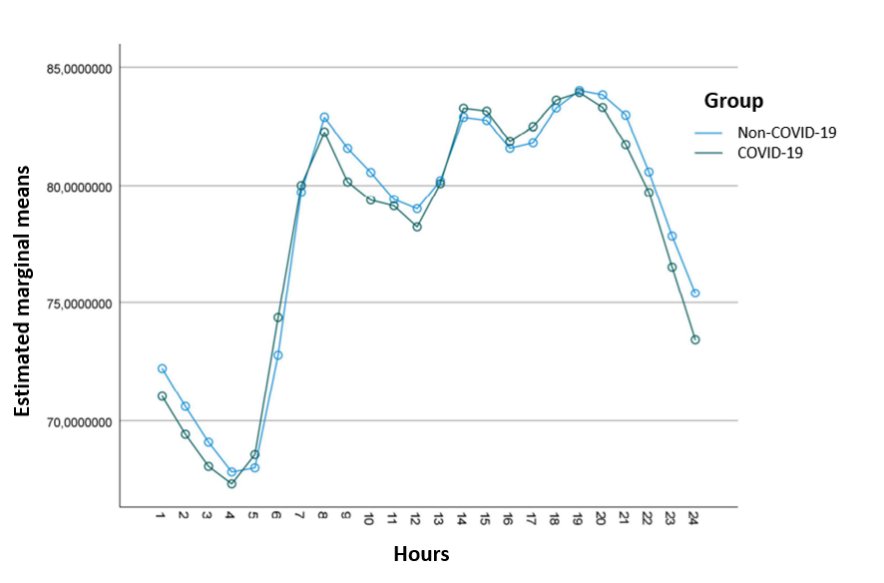
Average heart rate hourly activity curves for the COVID-19 and non–COVID-19 groups averaged across all volunteers in each group.

Figure 6 presents the overall shape of the mean heart rate curve along the day for both groups (averaged across all volunteers in each group). In general, both curves show a spike in heart rate activity at the beginning of work hours (around 6-8 h), another peak after lunch hours (13-14 h), and a steady decline during the nighttime (after 22 hours until the spike at 6 hours).

Using the MANOVA profile analysis, it was concluded that the level hypothesis cannot be rejected (*F*_1,74_=.107; *P*=.75), meaning that the baseline heart rate was similar across groups. However, the flatness hypothesis was rejected, as expected (Hotelling T²=20.282; *P*<.001). Finally, the parallel hypothesis was not rejected (Wilks Λ=0.697; *P*=.99), meaning that the curves presented the same pattern along the day, as confirmed by visual inspection.

Therefore, the smartwatch helped evaluate the COVID-19 and non–COVID-19 groups and enabled the conclusion that these groups did not differ in relation to their daily heart rate activity.

## Discussion

### Functional Model for Clinical Use of Data

The functional model presented in this study proved to be stable and ready to fulfill its role of receiving data from mobile devices and offering them for clinical use. At 35 weeks of follow-up, we did not have any interruption of its service, and it is being actively used to monitor the health of volunteers with >10 million records received by the platform and for >13,000 patient-days of clinical follow-up (Tables 5 and 6). With no intervention to encourage volunteers to synchronize the data, <0.5% of the records were synchronized in <1 hour, and only 5% of the records were synchronized in <2 hours (Table 8), indicating that this solution is not suitable for monitoring critical events and the alert system is unfit for urgent clinical use. However, two-thirds of the data were synchronized within 24 hours, and <10% of the data were synchronized in >7 days (Table 8), indicating that the digital solution is more than adequate to be used as a nonurgent health care tool.

Another relevant point of this model is that it follows all requirements of data protection and privacy laws. Data of interest for clinical follow-up are anonymously transmitted via the IoT through the study model’s own cloud and collected into the hospital’s data center, which is suited for processing health data according to the applicable laws. Furthermore, only within the hospital are the data complemented with other clinical and identification information, that is, the patient has the security and protection of their data collected by the wearables, and the physicians have access to the clinical data for monitoring.

Moreover, as a functional model platform developed with scalability and interoperability in mind, it is suitable to be used with IoT devices and other clinical software in multicenter, large-scale, and long-term follow-up studies, providing researchers and clinicians with a powerful tool to monitor their patients. In addition, because digital solutions are entirely based on cloud technology, they are accessible from any region of the world. Regions in need of medical assistance and located in hard-to-reach areas, such as Amazonas in Brazil, will be able to take advantage of this technology, as suggested in recent studies conducted by Hospital das Clínicas of the Faculdade de Medicina da Universidade de São Paulo [36]. These regions may greatly benefit from a system that enables patient follow-up remotely, such as the one proposed in this study.

### Manual Data Collection Methodology

For gold-standard equipment measurements, the protocols and recommendations are widely known from the literature. However, for novel technologies such as the smartwatch, not much is known about the methodology and best practices for clinical follow-up, and being subject to a learning curve was unavoidable during the study execution [37,38]. In this sense, a few possible confounders that could have not been controlled in this study (without prior knowledge) may have introduced noise in our results. Some of these should be investigated further in future studies to better understand how they alter the results, such as the best fit and location of the smartwatch on the wrist, the shape of the smartwatch’s surface that is in contact with the skin, and the use of dermatological products by the volunteers. In a similar study, Spaccarotella et al [39] highlighted the need for precise and repeatable smartwatch positioning and adjustment in the wrist of patients [39].

The simultaneous collection of BP on both wrists at the same time, following the instructions in the Galaxy Watch 4 manual, should also be reevaluated, because it introduces subjective variation related to the research monitor performing the tasks (such as synchronization of both measurements), which may influence the results obtained. The same-arm sequential collection (instead of opposite-arm simultaneous collection, as was performed) is also the most commonly used or recommended method in the literature for device comparison [40].

It is important to note that our study focused on smartwatch use on a regular day-to-day routine of volunteers to better reflect the real-life setup in which the system will be deployed. This means that we did not have an optimal preparation for data collection, nor did we have a controlled environment for acquisition, as is usually the case with other studies [41]. Furthermore, we had multiple gold-standard devices used for comparison and calibration, albeit being the same model; interequipment variation may have also introduced noise in the analysis. Similarly, because we had multiple data collectors (even for the same volunteer), additional external variability might have been introduced.

In the polysomnography data collection, it was found that some volunteers continued to sleep after the completion of the traditional polysomnography, identifying a possible failure in the collection methodology mainly related to the end of the examination, where the technique ended before the volunteer was fully awake. Therefore, despite successful data collection simultaneously with the gold-standard device, the examiner bias hindered the accurate analysis of the results.

### Galaxy Watch Features

The power of continuous data collection in a naturalistic manner brings immeasurable benefits to medical monitoring. In this study, this technology helped diagnose asymptomatic bradycardia in one of the volunteers whose only complaint was tiredness. Through the digital solution, the physician identified recurring moments of bradycardia outside medical care moments and was able to build a heartbeat variation curve throughout the day, which will certainly be very useful for monitoring many diseases.

### About Accuracy and Precision

The accuracy and precision study demonstrated that the measurement of heartbeats is above expectations, added to the continuous measurement technology, making it a highly valuable tool for telemonitoring patients [37]. The data indicate that oxygenation is suitable for use in clinical follow-up and remote monitoring. As it occurs in gold-standard equipment, measurements outside the clinical context (eg, oxygenation <88 with a eupneic volunteer) suggest an error, and a new measurement is suggested in any type of medical device [37]. Thus, the results indicate that these measures can support health care professionals in decision-making. If the oxygen saturation be improved with continuous measurement feature, such as heartbeat, the potential benefit of that would be of great value for remote monitoring.

As for BP, the data and observations mentioned in the collection methodology suggest that more studies are needed, especially for the validation method [38]. Unlike other studies, where measurements with wearables are performed in the laboratory, hospital conditions, or under observation, this study focused on day-to-day, real-life use of the smartwatch. Therefore, optimal conditions, as suggested in the literature [39,42], were not guaranteed or expected. These limitations can be noted in the BP correlation analysis, which achieved only moderate correlation with the gold-standard measurements. Notwithstanding these points, our agreement and accuracy results are similar or better than those of other studies involving BP and oxygen saturation measurements with smart devices [38,39,43-45], which suggests an adequate performance even during real-life conditions. Furthermore, the agreement obtained with the gold standards allied to the synchronization reliability of the platform allows us to suggest and consider the use of the smartwatch and the digital solution to follow patients with chronic disease, who can be remotely monitored by health professionals with data collected continuously or under user action.

These data collected under real-life conditions can support the clinical decision of the team of caregivers, with a potential reduction in coming and going to health services, and because it is a widely accepted equipment, there will be greater adherence to use, as it is not characterized as assistive equipment for the disability conditions [46].

Regarding polysomnography, the data indicate great potential for usability, mainly because of the ease of adherence of the volunteers; however, based on the collection methodology, a new study with greater convenience for the participants is suggested. Nevertheless, the results obtained, allied to the comfort afforded to the patient during acquisition (compared with the more intrusive polysomnography equipment), are quite promising.

### About COVID-19

All the volunteers were grouped by disease (COVID-19) status and demographic characteristics, and no significant difference was found in our study. This means that there was no loss in the quality of measurements, even in patients with COVID-19. With regard to the heart rate activity group comparison, it is notable that all volunteers from the COVID-19 group were highly functional and had already returned to their daily work life. Therefore, one would not expect many differences in heart rate performance, as observed.

### User Experience

The use of smartwatch for monitoring was very well evaluated by the volunteers, where 69% (47/68) agreed that it felt embedded in their body (Table 12), which supports the choice of the device for continuous monitoring use. In addition, the user experience results indicate that smartwatches have wide acceptance as an accessory, without the stigma of a medical device that can cause discomfort in patients with chronic illnesses, such as some assistive equipment that characterizes a disability for observers. This is particularly important for adherence to a remote monitoring program, as the use of smartwatches by patients with chronic illnesses does not make them feel like they have a disability [46].

### Limitations

The method used to measure BP simultaneously on opposite wrists, following the product’s instruction manual, generated a confounding factor based on the subjectivity of the research monitor when starting the data collection on the smartwatch.

This study considered a home use environment for remote monitoring. The measurement conditions for accuracy and precision are not those recommended by high-precision studies such as ISO 81060-2:2018 [42]. In addition, as the study volunteers were drawn from a healthy population, most of the values recorded throughout the project were within the normative range, which may also skew the results found, and is a limitation of our recruitment methodology. Calibration and manual collection of BP were performed using different digital devices. Despite being the same model and same brand, it became a confounding factor in the data analysis. Furthermore, there was no calibration of the gold-standard digital BP device throughout the study, simulating domestic use. A booklet of good practices for using the smartwatch for measurement is suggested to the manufacturer based on the experience of this study.

During polysomnography, the technician needed to end the procedure before the end of the volunteers’ sleep cycle, which became a limiting factor for the analysis regarding the end of the volunteers’ sleep.

### Cost

A total of US $39,053.98 (Brazilian real to US $ conversion of 5.80, obtained on December 31, 2021) were spent on smartwatches, gold-standard devices, and mobile phones in this study (Table 26).

**Table 26:** Total investment on devices and equipment.

Device	Quantity, n	Investment (US $)
Samsung Galaxy 4 smartwatch	84	19,828.73
Noninvasive blood pressure monitor	92	1866.57
Pulse oximeter for noncontinuous monitoring	92	1130.24
Samsung Galaxy smartphone A52	45	16,228.44

### Future Perspectives

The digital solution LIKA is currently a functional model composed of automation components [47] that could become a product that plays the role of a bridge between devices that use the IoT and health services, or as an increment for products that already exist. Compared with similar recent studies by Quer et al [12] and Mishra et al [13], which have approached the detection of COVID-19 through the use of heart rate, steps, and sleep data, the digital solution platform offers researchers and clinicians data collected in a naturalistic and continuous way. With the growth in the use of artificial intelligence in health care, the functional model presented will allow the formation of large databases for increasingly precise medicine [48]. Furthermore, a secure way of transmitting and receiving sensitive data, as done in this study, will allow predictive machine-learning models to offer preventive advice in real time to patients and clinicians, as many of these models are not yet available on mobile or wearable devices and need to be executed on dedicated hardware. However, the advent of highly sophisticated large language models will further improve patients’ health care, because wearable devices will be able to better communicate to (and receive requests from) the patient while gathering their vital signs for processing.

To better understand the role of smartwatches in telemonitoring patients in Brazil, a cost-effectiveness study could clarify the real benefits that wearable devices can offer by measuring vital signs in a naturalistic way. Furthermore, a follow-up study with patients or volunteers from different locations is necessary to prove the effectiveness of this functional model in different connectivity realities, because the proposed model relies on internet connection.

As very well detailed by Vijayan et al [24], smartwatches have a very wide applicability in the field of health care. As the digital solution was developed together with the manufacturer, it is ready to receive and provide, for clinical use, data from other devices not covered in this version of the study, such as the electrocardiogram for monitoring atrial fibrillation, reported in the studies by Nasarre et al [14], Bumgarner et al [15], and Perez et al [16].

### Conclusions

On the basis of the results obtained, considering the validation conditions of accuracy and precision, and simulating a home environment for layperson use, we conclude that the functional model built in this study, named LIKA, is able to capture data from the smartwatch and anonymously provide the data to health services, where they can be treated in accordance with legislation and can be used to support clinical decisions during remote monitoring.

## Abbreviations

ANVISA: Agência Nacional de Vigilância Sanitária
BP: blood pressure
bpm: beats per minute
IoT: Internet of Things
LGPD: Lei Geral de Proteção de Dados
LoA: limits of agreement
MANOVA: multivariate ANOVA
REDCap: Research Electronic Data Capture
REM: rapid eye movement
SE: sleep efficiency
TIB: time in bed
TR: tolerance region
TST: total sleep time
WASO: wake after sleep onset

## Data Availability

The data sets generated during or analyzed during this study are available from the corresponding author upon reasonable request.
